# When the rainy day is the worst hurricane ever: the effects of governmental policies on SMEs during COVID-19

**DOI:** 10.1007/s11187-021-00510-8

**Published:** 2021-05-18

**Authors:** Yacine Belghitar, Andrea Moro, Nemanja Radić

**Affiliations:** Cranfield School of Management, Cranfield University, College Rd, Cranfield, Bedford, MK43 0AL UK

**Keywords:** COVID-19, Cash Small firms, Economic crisis, Government intervention, D70, E65, G33, H12, L20, L26

## Abstract

We investigate the impact of COVID-19 on 42,401 UK SMEs and how government intervention affects their capability to survive the pandemic. The results show that, without governmental mitigation schemes, 59% of UK SMEs report negative earnings and that their residual life is reduced from 164 to 139 days. The analysis shows that government support scheme reduces the number of SMEs with negative earnings to 49% and allows extending the residual life for SMEs with negative earnings to 194 days. In addition, the support scheme reduces the number of jobs at risk in our sample by around 20%. However, our results suggest that weaker firms benefit more than strong ones. Besides, industries that are worst hit by COVID-19 are not those that benefit most from the government support scheme. We ascribe this result to the fact that the schemes do not discriminate between those firms that deserve support and those that do not deserve it.

## Introduction

The economic crisis linked to COVID-19 is very different from the last financial crisis (2007–2008) since it does not have a financial origin (bubble-burst cycle); it is the kind of black swan event that took the world economy by complete surprise (Aliber & Kindleberger, 2015; Beltratti & Stulz, 2012; Fitoussi & Saraceno, 2010; McGuinness & Hogan, 2016; Samatas et al., 2019). Besides, the consequences are dramatic: the USA expects a contraction of nearly 11% in terms of real GDP (Baker et al., 2020), with small businesses that are particularly affected since they are typically financially fragile (Bartik et al., 2020; Fairlie & Fossen, 2021); in the UK, according to a recent McKinsey survey of small and medium enterprises (SMEs), nearly 60% of firms surveyed believe they will be out of business by for April 2021 (Albonico et al., 2020). Indeed, the decision taken by many governments to confine people to stay in their houses to save lives has led to an acute cash crisis for many firms across different sectors. Firms operating in sectors such as tourism, hospitality, entertainment, and air transportation were particularly hard hit with their sales that are almost non-existent (ONS 2020). Leisure, entertainment, pubs, restaurants, and gyms are forced to close their operations overnight (Wiggins & Hancock, 2020). Travel limitations (allowed only for key workers) force airlines to ground entire fleets and train operators to reduce their activity to the bare minimum running trains almost empty (FT Reporters, 2020). In a similar vein, the cash position of consumer goods and retail firms is affected because of the reduced demand from households that are reshaping their spending habits (Wisniewski et al., 2021) and cutting all non-essential spending because of the fear that the economic crisis can adversely affect their jobs and future income (Fairlie & Fossen, 2021). Moreover, supply chain disruptions are so pervasive that even those firms that enjoyed a solid or increasing demand in their products (e.g. biotechnology firms) are adversely affected because they were not able to satisfy the demand. As a consequence, a larger number of firms are not able to generate the cash they needed to cover the operations.

Past studies provided different motives to why firms should hold cash. Within the precautionary motive, firm should hold cash to meet unexpected contingencies (Bigelli & Sánchez-Vidal, 2012). Almeida et al. (2011) documented that firms with little cash reserves are less likely to survive a crisis. Evidence from 2007 to 2008 financial crisis suggests that the probability of a firm’s survival depends largely on the level of cash held within its balance sheet (Kneer et al., 2019). The precautionary motive also suggests that firms with lower and more volatile cash flows, lower net working capital, higher R&D expenditure, and higher growth opportunities are more affected by a crisis and hence should hold more cash (Dittmar & Duchin, 2010; McLean, 2011; Opler et al., 1999). While the extant cash holdings literature deals mainly with why firms hold cash, nothing is known about how long a firm can survive with its cash reserves in the midst of a crisis, like the COVID-19. Against this background, we address the following research question: how long an SME can survive the unexpected COVID-19 shock given its cash reserves together with the provided UK government support? In so doing, we propose a novel forecasting approach based on firm’s earnings and cash reserves, in which sales shock and government supports are key inputs, to estimate the “residual life” of an SME under different scenarios. We are interested in finding out for how long an SME can survive the unexpected adverse shock linked to COIVID-19 given the cash it is able to generate and the cash it holds, and how the policies implemented by the UK government to support firms help SMEs to navigate the crisis by expanding the cash they can rely on. We perform our analysis by exploring the dynamics on a sample of 42,401 UK SMEs across 28 industries. Besides, since COVID-19 is having different impact for different industries and different regions, we explore its impact both at industry and on regional level.

To navigate through COVID-19’s hurricane is particularly difficult for SMEs for a different set of reasons. First, even if they are considered to be more flexible because of the flat organisational structure (Dean et al., 1998; Walker & Petty, 1978), in fact they are quite often characterised by a cost structure that is more rigid than larger organisations: even a small drop in sales can have an important impact on how efficiently the assets are used; the impact on the overall cost of each member of the staff is very relevant so that the decision to retain staff when sales temporarily decline can have a relevant negative impact on profitability. Thus, abrupt changes can adversely affect them (Ang, 1992; Ang et al., 1995). Second, SMEs have limited cash reserves, also because entrepreneur’s decision to pursue certain business is not essentially linked to profit maximisation (Hamilton, 2000; Kahneman, 2012; Kahneman et al., 1982; Moskowitz & Vissing-Jørgensen, 2002). Moreover, SMEs are not necessarily able to generate high returns in good times to protect them during economic downturns (Ang et al., 1995; Kahl et al., 2003; Moskowitz & Vissing-Jørgensen, 2002). Thus, a dramatic and sudden loss in the revenue and the related cash inflow can severely affect their ability to function as shown by empirical evidence (Ang, 1991, 1992; Cressy, 2006; Ghosal & Ye, 2015) and further confirmed in the case of the COVID-19 crisis by empirical evidence from Northern Italy (Organisation for Economic Cooperation & Development, 2020), the UK (Albonico et al., 2020) and the US (Fairlie & Fossen, 2021). Third, previous studies show that SMEs tend to hold low levels of cash compared to large firms because of their reduced dimension (Belghitar & Khan, 2013; Myers & Majluf, 1984). The consequence is that (fourth) they rely more on debt, leading to higher financial risk that can be unbearable during crises (Cassar & Holmes, 2003; Hutchinson, 1999) particularly when the cash inflows dry up and they struggle to access credit needed (Carbó‐Valverde et al. 2016; Casey & O’Toole, 2014; Moro et al., 2015). All in all, the above discussion suggests that the cash position of SMEs will be more acute to the COVID-19 crisis.

Emerging research focusing on COVID-19 finds that SMEs are deferring investments, reducing labour costs or reducing expenses (Thorgren & Williams, 2020), are increasing the use of bootstrap financing measures to mitigate the negative consequence of the crisis (Block et al., 2021), and are facing a significant reduction on entrepreneurial and innovative activity (Brown et al., 2020). Moreover, research suggests a gender discrimination with women-led firms that are more adversely affected by COVID-19 than men-led ones (Graeber et al., 2021). Thus, it is not a surprise that policy makers have been asked to take actions to support them to deal with the crisis. In spring 2020 the UK launched two supporting schemes: The Coronavirus Job Retention Scheme (CJRS) and the Bounce Back Loan Scheme (BBLS). The CJRS scheme allows the firm to avoid frictional costs linked to making employees redundant (e.g. the notice period) by furloughing employees so that the government covers 80% of their usual monthly wage up to £2500 a month. Any entity with a UK payroll can apply. The original scheme has been extended twice, and at the time of writing, it is expected to last until 30 April 2021.^1^ BBLS scheme provides financial support to businesses across the UK by helping SMEs to borrow between £2000 and up to 25% of their turnover via a government guarantee for the entire loan up to £50,000 so that firms can access credit in a period when banks are not keen to lend because of borrowers deteriorating performance. Both schemes apply to UK-based firms from any sector (except banks, insurers and reinsurers, public-sector bodies, and state-funded primary and secondary schools^2^) established before 1 March 2020 and that, at the time it submits its application, were not into (1) collective insolvency proceedings, (2) received restructuring aid or subject to a restructuring, and (3) in bankruptcy or liquidation. The UK scheme is different from other European schemes such as the French one that grants SMEs with turnover below €1 million and drop in turnover of at least 70% a €1,500 monthly compensation, or the German one that provides direct subsidies to one-person businesses and micro-enterprises by relying on a €10 billion fund (Organisation for Economic Cooperation & Development, 2020).

Past research suggests that policies aimed at facilitating access to finance and reducing the cost of borrowing can be helpful for firms in the short run (Duarte et al., 2018) and that the supporting policies (such as credit guarantees systems) may be effective in protecting the economy from collapsing (Yamori, 2015), as they allow firms to access the cash that they need. However, it is also pointed out that these supporting schemes can have a negative effect by discouraging firms from improving their efficiencies (Yamori, 2015).

Our results show that government COVID-19 mitigation policies seem to be quite successful in supporting small and medium sized firms. The policies have three main effects: (1) they reduce the number of jobs that would have been lost; (2) they reduce the number of SMEs with negative EBITDA; and (3) they extend the life of those SMEs that are burning cash. Thus, at first sight, the results suggest that the policies are successful in protecting the economy. However, our analysis also suggests that the SMEs that benefit the most in terms of extended days of life are those that are weak irrespective of COVID-19 effect. Besides, when we focus on the regional impact, we find that the biggest beneficiary of government intervention is the London area, even if the areas that are facing the greater negative economic impact by COVID-19 are Scotland and Northern Ireland. All in all, our evidence suggests that these two schemes overprotect weak firms and are not able to target industries/areas that are worst hit. We argue that this happens because the interventions are very general and are not able to target those firms that deserve support.

## Theoretical considerations and empirical framework

The concept of cash reserves (or holdings) can be traced back to Keynes (1936), who suggests three main motives for holding cash: (1) the transaction motive, need of cash to realise current transactions; (2) the precautionary motive, the need of cash to provide security with respect to unforeseen future events; and (3) the speculative motive, the need of cash to take advantage of unforeseen opportunities (Saeed et al., 2015). The precautionary motive perhaps is the most relevant in a period of crisis when analysing the liquidity of the firm. It suggests that cash reserve is considered as a valuable buffer to meet unexpected contingencies (Bigelli & Sánchez-Vidal, 2012).

As discussed at the outset of this paper, the crisis linked to COVID-19 is mainly the result of the forced shut down of the economies because of the lockdown. This event halted the production and the services provided by many firms so that they were not able to offer their products/services anymore (possibly the most dramatic example are the leisure, travel, and entertainment industries). Besides, those firms that were allowed to maintain their production were adversely affected by the disruption of their supply chains and the constrains linked to the access of inputs. In such a context, firm’s capability to generate cash is essential in order to live and in particular to navigate during the crisis time. When the firm has a positive cash flow, it should be able to maintain the current operations since it generates enough cash to cover all its immediate costs; on the other hand, when the expected cash flow is negative, the SME is implicitly “burning cash”. In this case, the “residual life” of the SME depends on the burning rate that is how rapidly the negative cash flow consumes the cash holdings that the SME can use in order to cover the current excess costs (cash outflow). Actually, SMEs can rely on cash in bank and any other very liquid asset, but also on debtors that are expected to be converted in cash quite soon.

In order to explore SMEs’ capability to survive COVID-19, we develop a simple but versatile forecasting model based on the firm EBITDA, in which sales shock and government supports are key inputs. Our model builds on the shock that firms face on their sales and then see how it affects the capability of the firms to generate positive EBITDA. By relying on EBITDA, we follow past research (Altman, 1968; Altman et al., 2012, 2020; Altman & Sabato, 2007; Mramor & Valentincic, 2003; Philosophov & Philosophov, 1999, 2005; Pompe & Bilderbeek, 2005; J. Thomas & Evanson, 1987) that argues that EBITDA is a measure of firm’s capability to generate operating margin and that it is a good proxy for the cash generated by the firm. Besides, SMEs tend to disclose aggregated data so that it is not possible to estimate precisely the cash flow.

We estimate EBITDA and then the “residual life” of the SME under three different scenarios:

- Ordinary scenario – expected EBITDA based on the past performance and not considering any negative effect linked to COVID-19 shock
- Scenario with the shock on sales at industry level as generated by COVID-19 – Office of National Statistics estimated reduction (ONS 2020)
- Scenario with the implementation of the UK government policies to support small firms (namely, the CJRS – also known as furlough scheme and similar to Italian “Cassa Integrazione Straordinaria” programme that was established in the 1951 and the hugely modified in 1975 or the “Kurzarbeit” programme that was originally established in 1910 in Germany) – and the BBLS

Our estimations are based on data up to 2018 in order to rely on a large dataset of 42,401 observations. Then, we re-estimate the three scenarios using 2019 data (the sample reduces to 18,626 observations).

### The calculation of the expected EBITDA

We assume the SME-specific relationship between change in sales, change in cost of goods sold (COGS), and change in cost of labour. We work out separate ratios because: (1) COGS and labour-related costs have different sensitivities to sales depending on how the firm runs operations (insourcing vs outsourcing; the role of raw material vs the role of labour; the capital intensity vs labour intensity of the production process; etc.) and (2) the UK governments launched a job specific scheme – the CJRS – that covers the cost of furloughed employees during the crisis period. Thus, in order to appreciate the real impact of this scheme, it is mandatory to keep the evolution of labour-related costs in isolation.

First, we estimate the sensitivity $\rho$ of change in COGS to change in sales for each year in the 3-year time window. Then, we average the two variations to obtain ${\rho }_{i,k}$ that is the sensitivity of the change of the COGS to change is sales of the SME *k,* operating in the industry *i*. Thus, the sensitivity is given by:

1 $${\rho }_{i,k}=\frac{1}{2}\sum_{t=1}^{3}\frac{({COGS}_{i,k,t}-{COGS}_{i,k,t-1})}{{(S}_{i,k,t}-{S}_{i,k,t-1})}$$

where ${S}_{i,k,t}$ are the sales and ${COGS}_{i,k,t}$ are the cost of goods sold of the SME *k* in industry *i* in year *t*. In a similar way, we work out the SMEs’ specific labour sensitivity ${\omega }_{i,k}$ that is the change in labour costs to change in sales of the SME *k* operating in the industry *i* as:

2 $${\omega }_{i,k}=\frac{1}{2}\sum_{t=1}^{3}\frac{{WORK}_{i,k,t}-{WORK}_{i,k,t-1}}{{S}_{i,k,t}-{S}_{i,k,t-1}}$$

where *S, t*, *k,* and *i* are defined as above, and $WORK$ are the labour costs.

We then estimate the SME specific expected change in sales for the year 2020 without COVID-19 by using the average change in sales for each SME for the 3-year period as follows:

3 $${\sigma }_{(i,k)}=\frac{1}{2}\sum_{t=1}^{3}\frac{{S}_{t}-{S}_{t-1}}{{S}_{t-1}}$$

where *S, t*, *k,* and *i* are defined as above. By relying on these sensitivities and the sales growth rate, we estimate the expected EBITDA for each SME in the case the operations were not affected by any shock as:

4 $${E(EBITDA)}_{\left(i,k\right) ORD}=\left(1+{\sigma }_{\left(i,k\right)}\right){S}_{\left(i,k,L\right)}-\left({COGS}_{\left(i,k,L\right)}+{\rho }_{i,k} {\sigma }_{\left(i,k\right)}{S}_{\left(i,k,L\right)}\right)-\left({WORK}_{\left(i,k,L\right)}+{\omega }_{i,k} {\sigma }_{\left(i,k\right)}{S}_{\left(i,k,L\right)}\right)$$

where $(1+{\sigma }_{\left(i,k\right)}){S}_{(i,k,L)}$ is the estimation of the expected sales of the SME *k* belonging to industry *i* based on the most recent year *L*, ${\rho }_{i,k}{\sigma }_{\left(i,k\right)}{S}_{(i,k,L)}$ is the estimate of the change in COGS linked to the increase in sales, and ${\omega }_{i,k} {\sigma }_{\left(i,k\right)}{S}_{(i,k,L)}$ is the estimate of the change in labour costs linked to the expected change in sales. The values resulting from this estimation are considered the base case under no COVID-19 shock (ORD).

For each SME, we can estimate the days of residual life ${\lambda }_{ORD}$ according to the following rule:

5 $$\left\{\begin{array}{c}{E(EBITDA)}_{\left(i,k\right) ORD}>0\Rightarrow {\lambda }_{\left(i,k\right) ORD}= \infty \\ {E(EBITDA)}_{\left(i,k\right) ORD}<0\Rightarrow {\lambda }_{\left(i,k\right) ORD}=-\frac{{\uptau }_{\left(i,k\right) }}{{E(EBITDA)}_{\left(i,k\right) ORD}}\end{array}\right.$$

where $\uptau ={\mathrm{Cash holdings}}_{i,k,L}+\beta {\mathrm{Creditors}}_{(i,k,L)}$ and $\beta$ represent the percentage of the non-delinquent customers. In the basic scenario, (ORD) $\beta$ is set to 1 (i.e. we assume no delinquency since the delinquency ratio of the years before COVID-19 is very small and limited to few firms), so that $\uptau$ represents the residual cash available to the SME. If the ${E(EBITDA)}_{\left(i,k\right) ORD}$ is positive, the SME is able to generate enough cash and will not consume cash holdings (if anything, it increases them), while in the scenario of negative ${E(EBITDA)}_{\left(i,k\right) ORD},$ the SME burns cash and has a residual limited life which depends on the burning rate and the cash available (cash holdings). The expected residual days are given by ${\lambda }_{\left(i,k\right) ORD}$.

### The impact of COVID-19

In order to explore the impact of the current pandemic, we proceed by introducing the industry-specific COVID-19-related shock ${\delta }_{i}$ that captures sales reduction for each industry as estimated by the Office of National Statistics and released on 14 July 2020 (ONS 2020). We scale the SME specific expected change in sales by ${(1-\delta }_{i})$. The new estimation of the EBITDA is therefore given as follows:

6 $${E(EBITDA)}_{Covid}={(1-\delta }_{i})(1+{\sigma }_{\left(i,k\right)}){S}_{(i,k,L)}-{(1-\delta }_{i})({COGS}_{(i,k,L)}+{\rho }_{i,k} {\sigma }_{\left(i,k\right)}{S}_{(i,k,L)})-{(1-\delta }_{i})({WORK}_{(i,k,L)}+{\omega }_{i,k} {\sigma }_{\left(i,k\right)}{S}_{(i,k,L)})$$

where ${(1-\delta }_{i})(1+{\sigma }_{\left(i,k\right)}){S}_{(i,k,L)}$ represents the new expected level of sales for the SME *k* in industry *i* due to shock linked to the change of sales ${\delta }_{i}$ in the in industry *i*, ${(1-\delta }_{i})({COGS}_{(i,k,L)}+{\rho }_{i,k} {\sigma }_{\left(i,k\right)}{S}_{(i,k,L)})$ is the new level of COGS, and ${(1-\delta }_{i})({WORK}_{(i,k,L)}+{\omega }_{i,k} {\sigma }_{\left(i,k\right)}{S}_{(i,k,L)})$ is the new level of labour-related costs.

Also, in this case, we estimate the days of residual life for the ordinary scenario using the below rule:

7 $$\left\{\begin{array}{c}{E(EBITDA)}_{\left(i,k\right) COVID}>0\Rightarrow {\lambda }_{\left(i,k\right) COVID}= \infty \\ {E(EBITDA)}_{\left(i,k\right) COViD}<0\Rightarrow {\lambda }_{\left(i,k\right) COVID}=-\frac{{\uptau }_{\left(i,k\right) }}{{E(EBITDA)}_{\left(i,k\right) COVID}}\end{array}\right.$$

We adjust for the economic shock by keeping into consideration that customers are trying to retain as much cash as possible. Since firms face a stop/reduced activity for around 3/3.5 months (around 30% of the year), we assume that they face a similar lack of cash that implies a similar percentage in terms of debtors’ default. Needless to say, this is an estimate. However, it is also important to point out that any different percentage in terms of payments received/delinquency has a proportional effect on the lack of cash and variation of the residual days.

### The mitigating role of government intervention

There are two major policies implemented by the UK government to support businesses: CJRS and BBLS. By covering labour costs, the CJRS allows the firm to retain their staff. In addition, the scheme allows the firm to avoid frictional costs linked to making employees redundant (e.g. the notice period and dismissing costs). We estimate the improvement in the EBITDA linked to CJRS to be as follows:

8 $$CJRS={(1-\delta }_{i})\left({WORK}_{\left(i,k,L\right)}+{\omega }_{i,k} {\sigma }_{\left(i,k\right)}{S}_{\left(i,k,L\right)}\right)-\left(\frac{{WORK}_{\left(i,k,L\right)}}{{S}_{\left(i,k,L\right)}}\right){(1-\delta }_{i})(1+{\sigma }_{\left(i,k\right)}){S}_{(i,k,L)}$$

where ${(1-\delta }_{i})({WORK}_{(i,k,L)}+{\omega }_{i,k} {\sigma }_{\left(i,k\right)}{S}_{(i,k,L)})$ is the cost of labour under COVID-19 that consider SME’s rigidities in adjusting to the reduced sales, and $\left(\frac{{WORK}_{\left(i,k,L\right)}}{{S}_{\left(i,k,L\right)}}\right){(1-\delta }_{i})(1+{\sigma }_{\left(i,k\right)}){S}_{(i,k,L)}$ is the (theoretical) cost of labour the SME would have incurred without any friction by making employees redundant. This implies that the ${E(EBITDA)}_{\left(i,k\right) CRJS}$ is the corrected EBITDA taking into consideration the benefit of CJRS, and it is expressed as follows:

9 $${E(EBITDA)}_{CJRS}={E(EBITDA)}_{Covid}+CJRS$$

We estimate the days of life with CJRS according the rule:

10 $$\left\{\begin{array}{c}{E\left(EBITDA\right)}_{CJRS}>0\Rightarrow {\lambda }_{CJRS}=\infty \\ {E\left(EBITDA\right)}_{CJRS}<0\Rightarrow {\lambda }_{CJRS}=-\frac{{\uptau }_{\left(i,k\right) }}{{E(EBITDA)}_{CJRS}}\end{array}\right.$$

As far as the BBLS, the government intervention consists in underwriting bank loans up to 50,000 GBP. The rationale of this intervention is to grant credit access to small firms in a period when banks are not keen to lend because of the deteriorating performance of SMEs and the increased risk of default on loan repayments. BBLS impact is not in terms of cost reduction but in terms of increased cash holdings. To explore BBLS, we adjust the survival days and use to the following rule:

11 $$\left\{\begin{array}{c}if {E(EBITDA)}_{CJRS+LOAN}>0\Rightarrow {\lambda }_{CRJS}=\infty \\ if {E(EBITDA)}_{CJRS+LOAN}<0\Rightarrow {\lambda }_{CRJS+BBLS}=-\frac{{\uptau }_{\left(i,k\right) }+\mathrm{BBLS}}{{E(EBITDA)}_{CJRS}}\end{array}\right.$$

so that ${\lambda }_{CRJS+BBLS}$ gives the new estimation of residual days for SMEs thanks to the extra covered costs and the extra cash available because of both government schemes.

### Data

Our analysis relies on UK data about SMEs with sales and total assets size between 10,000 and 50,000,000 GBP and a number of employees up to 250. Thus, our sample of SMEs is in line with the definition of small and medium enterprise according to the EU standards (European Commission 2003). We use firm-level data obtained from the Fame – Bureau van Dijk – database for the fiscal years 2016–2019. To draw meaningful inferences, we require each SME to have at least 3 years of data. We exclude SMEs classed as public administration, education, health social services (BvD sector), guarantee (legal form), and SMEs without employees. Overall, this results in an original sample size of 42,401 firm observations across 28 industries in the UK.

Table 1 provides SMEs key descriptive statistics for the main variables used in the empirical framework. We find that, on average, an SME has total assets of 7.3 million with 47 workers. Further, the average cash held in the bank is about £967,000 (13.3% of total assets), while on average, SME owes £1,370,000 (18.8% of total assets). Moreover, the average firm creditor claim is £804,000 (11% of total assets). Turning attention on the split dataset (weak vs solid ones) it is interesting to stress that even if weaker firms have averages for all the variables considered that are greater than the solid ones, nevertheless the differences are not different since both weak and solid firms subsamples are characterised by very large standard deviations.

**Table 1:** Descriptive statistics entire dataset

Variable	Mean	Std. Dev	Min	Max
Full sample,*n*: 42,401 SMEs^a^				
Sales	8042	9104	8.60	49,978
COGS	7471	7828	0.00	39,957
WORK	3799	4833	0.00	39,209
Debtors	1370	1730	0.00	9992
Cash in bank	967	1526	0.00	9993
Creditors	804	1229	0.00	9971
Employees	47	52	1.00	409
Total assets	7292	8307	10.00	62,769
Weak firms,* n*: 10,461 SMEs^b^				
Sales	9908	9702	10.88	49,467
COGS	9323	8288	0.00	39,957
WORK	5141	5162	0.00	39,209
Debtors	1726	1833	0.00	9,992
Cash in bank	1069	1566	0.00	9987
Creditors	1040	1367	0.00	9845
Employees	56	55	1.00	409
Total assets	8507	8077	10.25	49,704
Solid firms, *n*: 31,940 SMEs^c^				
Sales	7430	8801	8.60	49,978
COGS	6864	7522	0.00	39,916
WORK	3360	4635	0.00	39,054
Debtors	1253	1676	0.00	9982
Cash in bank	933	1512	0.00	9987
Creditors	727	1166	0.00	9971
Employees	44	51	1.00	297
Total assets	6894	8350	10.00	62,769

This table provides the summary statistics for the main variables used in the empirical framework. *Sales*, average amount of sales in the three years; *COGS*, average cost of goods sold (net labour costs) in the 3 years; *WORK*, average cost of labour in the 3 years; *Debtors*, average end of year debtors in the 3 years; *Creditors*, average end of year creditors in the 3 years; *Cash in* *bank*, average end of year cash in bank in the 3 years; *Employees*, average number of employees in the 3 years

^a^Full sample,*n*: 42,401 SMEs

^b^Weak firms, *n*: 10,461 SMEs

^c^Solid firms, *n*: 31,940 SMEs

## Results

Table 2 presents the results on the entire sample for the tree scenarios: ordinary scenario (expected situation without COVID-19), scenario that includes the impact of COVID-19 and the scenario that considers UK government intervention aid policies. We start by analysing the expected EBITDA and residual cash available for each SME. In the ordinary scenario, we find that the average expected EBITDA is positive (£1,77 million), suggesting that the average SME is able to generate enough margin to support its operations. The survival rate (that is the percentage of SMEs generating positive EBITDA) is 75%. Besides, those SMEs that are not able to cover the current operations and the related cash outflows with the cash inflows (that is, those SMEs that are burning cash) have on average 164 days of cash to cover the operations (ordinary scenario, rows 1–3).

**Table 2:** Results for the entire dataset

	Variable	Mean	Std. Dev	Min	Max
Ordinary	EBITDA_B	1769.14	4750.70	− 19,990.02	19,995.18
	SURVIVE_B	75.33%	0.43	-	1.00
	LIFE_DAYS_B	164	151	-	547
COVID-19	EBITDA_C	− 1048.06	3401.94	− 24,907.47	19,811.36
	SURVIVE_C	41.41%	0.49	-	1.00
	LIFE_DAYS_C	139	143	-	547
Gov’t mitigation policies	EBITDA_G	− 621.57	3087.05	− 19,997.38	19,811.36
	SURVIVE_G	51.30%	0.50	-	1.00
	LIFE_DAYS_G	183	152	-	547
	LIFE_DAYS_G + 25,000	189	150	0	547
	LIFE_DAYS_G + 50,000	194	149	1	547
Effects on jobs	Job_loss_B	10,461			
	Job_loss_C	24,843			
	Job_loss_G	20,650			

This table presents the results for tree scenarios: ordinary scenario, impact of COVID-19, and government intervention policies, and their impact on the performance and residual life of the firm for the entire dataset. The variables are defined as follows: *EBITDA_B*, estimate of the EBTDA under standard condition; *SURVIVE_B*, proportion of firms that have a positive estimated EBITDA; *LIFE_DAYS_B*, days of available cash to cover the negative EBITDA_B (the value is estimated only for those firms with a negative EBITDA_B; *EBITDA_C*, estimate of the EBTDA under COVID-1919 shock; *SURVIVE_C*, proportion of firms that have a positive estimated EBITDA under COVID-19; *LIFE_DAYS_C*, days of available cash to cover the negative EBITDA_C (the value is estimated only for those firms with a negative EBITDA_C); *EBITDA_G*, estimate of the EBTDA under including the effect of government mitigation policies; *SURVIVE_G*, proportion of firms that have a positive estimated EBITDA under government mitigation policies; *LIFE_DAYS_C*, days of available cash to cover the negative EBITDA_G (the value is estimated only for those firms with a negative EBITDA_G); *JOB_LOSS_B*, number of jobs at risk in the ordinary scenario (e.g. number of jobs linked to firms with a negative EBITDA_B); *JOB_LOSS_G*, number of jobs at risk in the COVID-19 scenario (e.g. number jobs linked to firms with a negative EBITDA_C); *JOB_LOSS_G*, number of jobs at risk in the scenario with mitigating policies (e.g. number jobs linked to firms with a negative EBITDA_G)

*n *42,401

Next, we focus on the two scenarios that include the shock/disruption. First, we estimate SMEs’ EBITDA according to the shock on sales at industry level as generated by COVID-19 (no government mitigation). Second, we estimate the EBITDA by including the two government mitigation policies (CJRS or BBLS). Focusing on the SMEs under COVID-19 scenario (rows 4–6), we find that the average firm in the sample is burning cash (EBITDA: − 1,05 million GBP) which is reflected via the dramatically lower “survival rate” that drops from 75 to 41%. Besides, the residual life for those SMEs that burn cash is now on average 139 days. When we include the government mitigation policies (rows 7–11), we find that the average EBITDA is still negative (− 621,000 GBP). Also, and as a direct result of government intervention, “residual life” for SMEs that burn cash increases to 183 due to CJRS, or (by also including BBLS) between 189 and 194 (depending on the amount of loan taken). All in all, the government intervention has two positive effects: (1) it reduces the number of SMEs with negative EBITDA by 10% (50.30 vs 41.41%), and (2) it extends the survival days of those SMEs with negative EBITDA (SMEs that are burning cash) by 50 to 60 days. Our results reflect what has been found in Germany (Block et al., 2020).

Lastly, we investigate the effects on the number of jobs that are at risk as a result of the economic shock. We estimate this by looking at the number of jobs associated to SMEs with negative EBITDA in different scenarios. We find that due to COVID-19 the number of jobs at risk in our sample increases to 24,843 (from 10,461 in ordinary scenario) but when the CJRS is taken into consideration so that labour costs are partially covered by the government, the jobs at risk decrease to 20,650 (17% decrease).

**Fig. 1:**
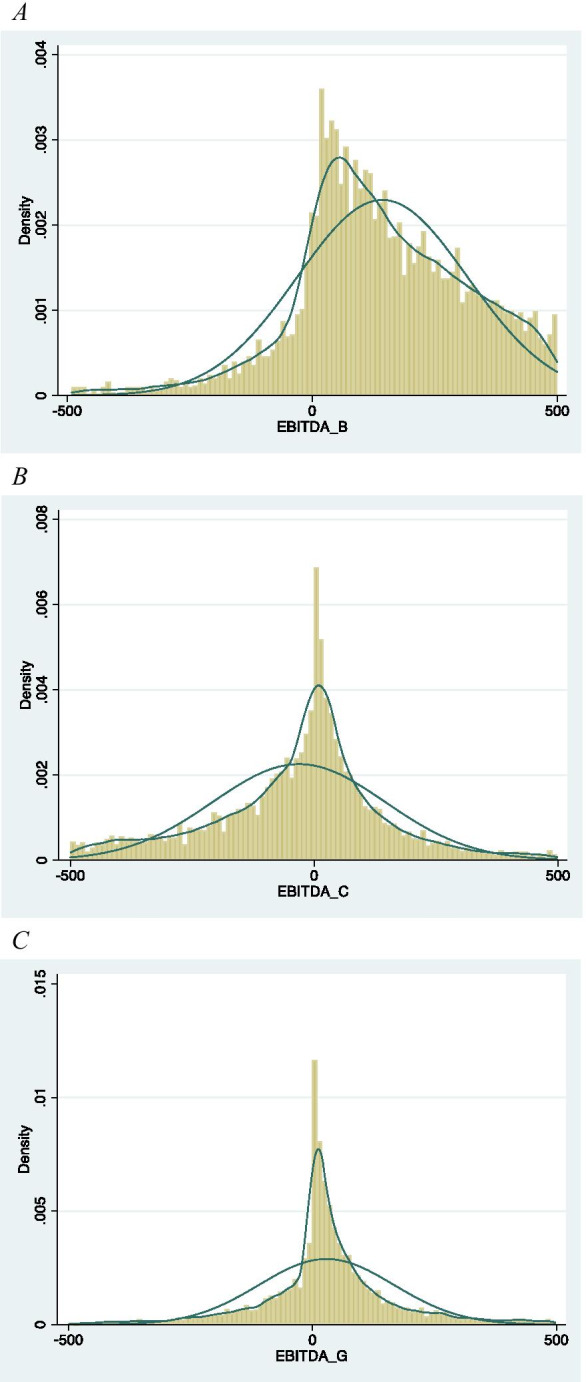
Distribution of the EBITDA under different scenarios. **A** Distribution of EBITDA in the ordinary scenario. **B** Distribution of EBITDA in the COVID-19 scenario. **C** Distribution of EBITDA with government mitigation policies scenario

Further evidence of the effect of COVID-19 and the policies implemented by the UK government emerges from Fig. 1A, B, and C.

COVID-19 moves the distribution of the expected EBITDA to the left, increasing dramatically the negative tail of the distribution. The government mitigation policies readjust the distribution moving it to the right. However, EBITDA distribution after the government intervention is more concentrated around the zero than the distribution without COVID-19.

Our next step is to explore the effect of COVID-19 on the vulnerable SMEs, that is SMEs with negative EBITDA in the ordinary scenario. The results are reported in Table 3.

**Table 3:** Weak firms – firms with expected negative EBITDA

	Variable	Mean	Std. Dev	Min	Max
Ordinary	EBITDA_B	− 2852.23	3451.38	− 19,990.02	− 0.01
	SURVIVE_B	0.00%	-	-	-
	LIFE_DAYS_B	164	151	-	547
COVID-19	EBITDA_C	− 3751.51	3961.70	− 24,907.47	-
	SURVIVE_C	0.00%	-	-	-
	LIFE_DAYS_C	119	132	-	547
Gov’t mitigation policies	EBITDA_G	− 2845.14	3566.87	− 19,997.38	16,184.65
	SURVIVE_G	8.38%	0.28	-	1.00
	LIFE_DAYS_G	186	151	-	547
	LIFE_DAYS_G + 25,000	191	149	0	547
	LIFE_DAYS_G + 50,000	194	148	1	547
Effects on jobs	Job_loss_B	10,461			
	Job_loss_C	10,461			
	Job_loss_G	9,584			

This table presents the results for tree scenarios: ordinary scenario, impact of COVID-19 and government intervention policies, and their impact on the performance and residual life of the firm focusing only on the SMEs with expected negative EBITDA. The variables are defined as follows*: EBITDA_B*, estimate of the EBTDA under standard condition; *SURVIVE_B*, proportion of firms that have a positive estimated EBITDA; *LIFE_DAYS_B*, days of available cash to cover the negative EBITDA_B (the value is estimated only for those firms with a negative EBITDA_B; *EBITDA_C*, estimate of the EBTDA under COVID-1919 shock; *SURVIVE_C*, proportion of firms that have a positive estimated EBITDA under COVID-19; *LIFE_DAYS_C*, days of available cash to cover the negative EBITDA_C (the value is estimated only for those firms with a negative EBITDA_C); *EBITDA_G*, estimate of the EBTDA under including the effect of government mitigation policies; *SURVIVE_G*, proportion of firms that have a positive estimated EBITDA under government mitigation policies; *LIFE_DAYS_C*, days of available cash to cover the negative EBITDA_G (the value is estimated only for those firms with a negative EBITDA_G); *JOB_LOSS_B*, number of jobs at risk in the ordinary scenario (e.g. number of jobs linked to firms with a negative EBITDA_B); *JOB_LOSS_G*, number of jobs at risk in the COVID-19 scenario (e.g. number jobs linked to firms with a negative EBITDA_C); *JOB_LOSS_G*, number of jobs at risk in the scenario with mitigating policies (e.g. number jobs linked to firms with a negative EBITDA_G)

*n* 10,461

In fact, these SMEs are expected to burn cash irrespective of COVID-19 and consequently to be at greater risk and have a shorter residual life. The average EBITDA of these firms in the sample is − 2,85 million GBP. Considering the cash holdings these SMEs have, their average expected residual life is 164 days (ordinary scenario, rows 1–3). During COVID-19 scenario (rows 4–6), the EBITDA goes down further (− 3,75 million GBP), so that the residual life reduces to 119 days (4 months). When we account for the government mitigation policies (rows 7–11), the average EBITDA improves and goes back to levels very close to those of ordinary scenario level (− 2,84 million GBP). Besides, the survival rate improves to 8.3%. Consequently, the residual life increases to 186 days due to CJRS, or via BBLS between 191 and 194 days, depending on the amount of loan taken. As far as jobs at risks are concerned, there is no difference between ordinary and COVID-19 scenarios (and this is expected as these SMEs have jobs at risk regardless). Thus, the only expected impact would be via CJRS for which the job losses decrease to 9584: by supporting weak SMEs, the governments’ mitigation policy allows SMEs to retain 877 jobs (protecting 8.4% of potential job losses)^3^ and extends firms’ life for a period longer than the average survival period these firm had without COVID-19.

We also estimate the impact on SMEs with a positive EBITDA (Table 4) in order to be able to compare the effect of the policies for strong and weak SMEs.

**Table 4:** Solid firms – firms with expected positive EBITDA

	Variable	Mean	Std. Dev	Min	Max
Ordinary	EBITDA_B	3282.73	4095.59	0.12	19,995.18
	SURVIVE_B	100.00%	-	1.00	1.00
	LIFE_DAYS_B				
COVID	EBITDA_C	− 162.63	2654.39	− 21,817.01	19,811.36
	SURVIVE_C	54.97%	0.50	-	1.00
	LIFE_DAYS_C	155	151	-	547
Gov’t Policies	EBITDA_G	106.69	2516.90	− 19,247.13	19,811.36
	SURVIVE_G	65.35%	0.48	-	1.00
	LIFE_DAYS_G	180	153	-	547
	LIFE_DAYS_G + 25,000	188	151	1	547
	LIFE_DAYS_G + 50,000	193	150	1	547
Effects of jobs	Job_loss_B	0			
	Job_loss_C	14,382			
	Job_loss_G	11,066			

This table presents the results for tree scenarios: ordinary scenario, impact of COVID-19 and government intervention policies; and their impact on the performance and residual life of the firm focusing only on the SMEs with expected positive EBITDA. The variables are defined as follows: *EBITDA_B*, estimate of the EBTDA under standard condition; *SURVIVE_B*, proportion of firms that have a positive estimated EBITDA; *LIFE_DAYS_B*, days of available cash to cover the negative EBITDA_B (the value is estimated only for those firms with a negative EBITDA_B; *EBITDA_C*, estimate of the EBTDA under COVID-1919 shock; *SURVIVE_C*, proportion of firms that have a positive estimated EBITDA under COVID-19; *LIFE_DAYS_C*, days of available cash to cover the negative EBITDA_C (the value is estimated only for those firms with a negative EBITDA_C); *EBITDA_G*, estimate of the EBTDA under including the effect of government mitigation policies; *SURVIVE_G*, proportion of firms that have a positive estimated EBITDA under government mitigation policies; *LIFE_DAYS_C*, days of available cash to cover the negative EBITDA_G (the value is estimated only for those firms with a negative EBITDA_G); *JOB_LOSS_B*, number of jobs at risk in the ordinary scenario (e.g. number of jobs linked to firms with a negative EBITDA_B); *JOB_LOSS_G*, number of jobs at risk in the COVID-19 scenario (e.g. number jobs linked to firms with a negative EBITDA_C); *JOB_LOSS_G*, number of jobs at risk in the scenario with mitigating policies (e.g. number jobs linked to firms with a negative EBITDA_G)

*n *31,940

In this case, the EBITDA under COVID-19 is marginally negative − 162,000 GBP. A closer look at the distribution of earnings shows that around 53% of the SMEs have negative EBITDA. The expected residual life of strong SMEs is 162 days. The government mitigation policies improve the EBITDA to positive 160,000 GBP, and the number of SMEs with positive EBITDA increases to 65%. In addition, the residual life of the SME with negative EBITDA is between 180 (CJRS only) and 193 days (CJRS and BBLS at 50,000 GBP). However, not all the firms that would have been profitable without COVID are profitable under the two governmental schemes since around 35% maintain a negative performance.

To further explore the SMEs’ capability to survive the pandemic, we perform two additional checks, focusing on different regions and on different industries (these results are shown in Tables 5 and 6).

**Table 5:** EBITDA effects of the COVID-19 in different regions

		Ordinary			COVID-19			Government mitigation policies				
Region	Obs	EBITDA	SURVIVE	LIFE_DAYS	EBITDA	SURVIVE	LIFE_DAYS	EBITDA	SURVIVE	LIFE_DAYS	LIFE_DAYS 25,000	LIFE_DAYS 50,000
Scotland	2673	1499.68	72.99%	167	− 1368.81	33.63%	131	− 959.31	44.78%	176	184.61	188.51
Wales	887	1061.65	69.56%	154	− 1226.67	34.27%	128	− 801.31	47.24%	171	180.63	186.55
Northern Ireland	1102	1335.87	73.68%	178	− 1069.62	38.11%	127	− 777.66	48.55%	148	155.78	164.07
North	7524	1792.85	74.95%	166	− 1133.33	39.61%	134	− 736.53	49.64%	181	185.85	190.50
Midlands	7156	1910.79	75.35%	166	− 1037.88	39.70%	130	− 659.43	49.59%	178	183.85	188.24
South East	6476	1852.49	77.41%	167	− 906.54	43.92%	141	− 533.33	54.12%	183	189.82	194.12
South West	3386	1570.02	73.60%	157	− 1175.37	39.75%	139	− 800.48	50.06%	174	182.06	185.00
London	10,971	1806.42	75.77%	162	− 974.71	45.18%	154	− 428.95	54.22%	204	209.76	215.40

*NB *BvD does not provide information about the region for 2226 firms. This table presents the estimates for EBITDA under the three different scenarios (ordinary scenario, impact of COVID-19, and government mitigation policies) focusing on different regions in the UK. The variables are defined as follows*: EBITDA*, earnings before interest depreciation and amortisation; *SURVIVE*, proportion of firms that have a positive estimated EBITDA under the three different scenarios; *LIFE_DAYS*, days of available cash to cover the negative EBITDA under the three different scenarios; *LIFE_DAYS 25,000*, residual days with BBLS at 25,000; *LIFE_DAYS 50,000*, residual days with BBLS at 25,000

**Table 6:** EBITDA effects of the COVID-19 in different industries

		Ordinary			COVID-19			Government mitigation policies				
Industry	Prop	EBITDA	SURVIVE	LIFE_DAYS	EBITDA	SURVIVE	LIFE_DAYS	EBITDA	SURVIVE	LIFE_DAYS	LIFE_DAYS 25,000	LIFE_DAYS 50,000
Agriculture	1.12%	1639.35	76.25%	129	− 836.05	45.75%	110	− 694.38	48.15%	134	141.27	149.02
Construction	7.34%	614.82	59.04%	170	− 2,217.05	31.99%	128	− 1,803.29	34.59%	177	181.35	185.46
Manufacturing	11.96%	2301.08	68.58%	179	− 1,612.42	31.11%	129	− 1,102.38	36.57%	177	181.82	185.95
Utility	2.67%	1752.02	74.79%	160	− 1,122.34	47.20%	131	− 858.30	49.77%	147	146.81	151.29
Trade	12.70%	3042.69	78.43%	133	− 92.65	50.19%	108	180.39	56.63%	156	166.16	172.51
Service	64.21%	1532.93	78.17%	164	− 960.91	43.01%	150	− 510.08	55.98%	195	202.27	206.91

This table presents the estimates for EBITDA under the three different scenarios (ordinary scenario, impact of COVID-19 and government mitigation policies) focusing on different industries. The variables are defined as follows: *EBITDA*, earnings before interest depreciation and amortisation; *SURVIVE*, proportion of firms that have a positive estimated EBITDA under the three different scenarios; *LIFE_DAYS*, days of available cash to cover the negative EBITDA under the three different scenarios; *LIFE_DAYS 25,000*, residual days with BBLS at 25,000; *LIFE_DAYS 50,000*, residual days with BBLS at 25,000

In our ordinary scenario (columns 3–5, Table 5), out of all the regions, SMEs in Wales have the lowest expected EBITDA (£1,06 million) with a survival rate of 69.5% and 154 days of available cash. SMEs in Midlands have the highest expected EBITDA (£1,91 million), while SMEs in South East have the highest survival rate of 77.4%. During COVID-19 scenario (with no government intervention, columns 6–8), SMEs in Scotland are the hardest hit on average with EBITDA of − 1,36 million GBP and a survival rate of 33.6%, while SMEs in Northern Ireland have the least cash days available (127 days). SMEs in London have the highest survival rate, 45.1%, and the longest expected residual life of those SMEs burning cash (154 days). Focusing on the government mitigation policies (columns 9–13), we find that both the EBITDA and the survival rate improve, when compared to the ordinary scenario. Survival rate improves across all regions with improvements between 9 and 13%. More specifically and focusing on CJRS only, SMEs have between 9 and 42 extra days, even compared to pre COVID-19 levels. The only exception here is Northern Ireland, which starts with 178 days of cash (during ordinary scenario) but ends with 148 days even after considering for CJRS. When considering the BBLS, the residual life increases further from CJRS only scenario from 4 to 9 days or 9 to 16 days (depending on the loan option taken by the SME). It is also clear from the analysis that the biggest beneficiaries of government intervention are South East and London, while worst hit areas are Scotland and Northern Ireland.

Table 6 shows the results of EBITDA effects of the COVID-19 for different industries.

In our ordinary scenario (columns 3–5, Table 6), SMEs in construction industry have the lowest expected EBITDA (£614,000) and a survival rate of 59%, while shortest residual life is in agriculture industry. SMEs in trade industry have the highest expected EBITDA (£3,04 million) and the highest survival probability of 78.4%. Manufacturing is the industry with the longest expected residual life for those SMEs burning cash (179 days). During COVID-19 scenario (with no government intervention, columns 6–8), construction industry continues to be the worst performer and hardest hit on average with EBITDA of − 2,22 million GBP and survival probability of 31%, while SMEs in the trade industry now hold only 108 days of cash. This finding is in line with the latest report from Bank of England on the expected impacts of COVID-19 by industry (Bank of England, 2020), where it is argued that consumer-facing businesses and non-food manufacturing and construction were the most affected sectors. Focusing on the government mitigation scenario (columns 9–13, Table 6), we find that both the average cash burning rate and the survival rate improve, and they are similar to the ordinary scenario. Survival probability improves across industries between 2 and 12%. More specifically and focusing on CJRS only, SMEs now hold between 16 and 48 additional days of cash, compared to COVID-19 levels. When considering the BBLS, the days of additional cash increase further compared to CJRS only scenario between 4 and 16 days (depending on industry and the loan option taken by the SME). We can also observe from our analysis that the biggest hit industries are agriculture and construction, while the biggest beneficiaries are SMEs in service industry.

### Robustness test

As a robustness check, we redo the analysis reported above using a smaller sample of 18,626 SMEs that have financial information for the year 2019. Results are reported in Table 7.

**Table 7:** EBITDA effects of the COVID-19 – 2017–2019 sample

Results on the entire dataset						
	Variable	Obs	Mean	Std. Dev	Min	Max
Basic	EBITDA_B	18,718	2128.65	5972.35	− 39,618.84	96,722.75
	SURVIVE_B	18,718	77.65%	0.42	-	1.00
	LIFE_DAYS_B	2250	171	151	-	547
COVID	EBITDA_C	18,718	− 3320.00	5830.00	− 56,631.00	83,463.00
	SURVIVE_C	9998	22.02%	41%	-	100%
	LIFE_DAYS_C	18,693	112	123	-	547
Gov’t Policies	EBITDA_G	18,693	− 1830.67	4609.45	− 42,820.38	83,463.15
	SURVIVE_G	18,718	41.48%	0.49	-	1.00
	LIFE_DAYS_G	6193	237	136	-	547
	LIFE_DAYS_G + 25,000	6124	240	134	1	547
	LIFE_DAYS_G + 50,000	6061	242	133	1	547
Effects of jobs	Job_loss_B	4183				
	Job_loss_C	14,594				
	Job_loss_G	10,953				

This table presents the estimates for EBITDA under the three different scenarios (ordinary scenario, impact of COVID-19 and government mitigation policies) focusing on different industries. The variables are defined as follows: *EBITDA*, earnings before interest depreciation and amortisation; *SURVIVE*, proportion of firms that have a positive estimated EBITDA under the three different scenarios*; LIFE_DAYS*, days of available cash to cover the negative EBITDA under the three different scenarios; *LIFE_DAYS 25,000*, residual days with BBLS at 25,000; *LIFE_DAYS 50,000*, residual days with BBLS at 25,000

The analysis based on more recent dataset suggests that the average EBITDA moves from the expected 2,128,000 without COVID-19 to − 3,320,000, and the number of firms having a positive EBITDA decreases from 77 to 22%. Also, the life expectancy for firms burning cash reduces from 171 to 121 days. Government intervention improves the average EBITDA that, nevertheless, remains in the negative. Besides, governmental intervention reduces the number of firms with negative EBITDA but only 41% of them have an expected positive EBITDA. If anything, the results based on more recent data suggest even a harsher effect of CONVID-19 as in the case of firms that turn to have a negative EBITDA (those with positive EBITDA used to be 41%), a smaller positive effect of the governmental intervention (the results using 2016–2018 data suggests that around half of the firm have a positive EBITDA). However, the differences are not statistically different.

All in all, the fact that the results of the robustness test are qualitatively similar to those of the original analysis confirms and reinforces our results in terms of huge negative impact and relatively reduced mitigation impact generated by the government intervention.

## Discussion and policy implications

The various strategies that have been implemented by the governments around the world (limiting people’s circulation, closing entire industries and limiting the operations of firms in a direct or indirect way) in order to deal with containing COVID-19 pandemic are affecting the economy of every country. Despite the intervention governments are implementing, there is a large decline in the economic activity.

Governments’ interventions to mitigate this economic shock seems to be mainly focused on allowing SMEs to survive the period of (forced) reduced activity and to retain jobs so that they will be able to jumpstart as soon as the economy reopens. Our evidence for UK SMEs suggests that the CJRS as well as BBLS address the problem but only to a certain extent. In fact, according to our estimate CJRS allows SMEs to cover some costs and protect cash as well as to retain around 17% of jobs that otherwise would have been lost. In addition, the joint effect of CJRS and BBLS allows SMEs with a negative EBITDA to extend their expected residual life by around 2 months (i.e. increase the life under COVID-19 by 35%) so that they can navigate the period of reduced activity and be ready when the economy will restart.

**Fig. 2:**
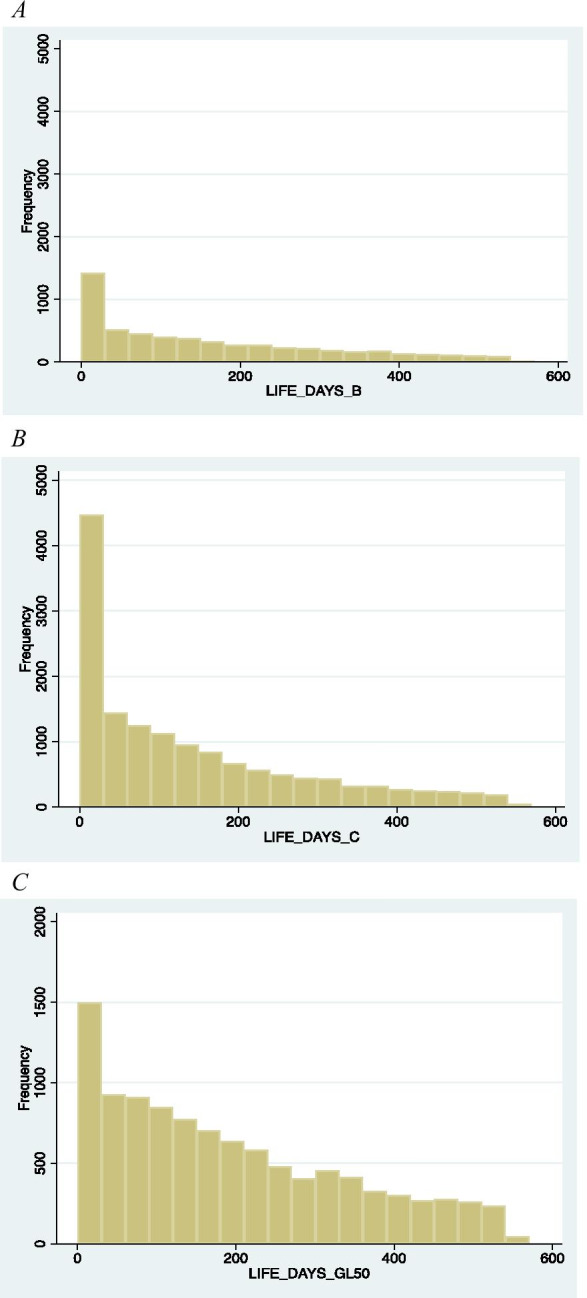
Distribution of the residual days under different scenarios. **A** Distribution of residual days in the ordinary scenario. **B** Distribution of residual days with COVID-19 scenario. **C** Distribution of residual days with government mitigation policies scenario

The policies implemented in the UK do not discriminate ex ante between strong and weak SMEs. Our comparative results suggest that by helping all the firms without selecting those that merit more support, government is able to protect around 8% of the jobs (that is to reduce job losses that are expected to occur under COVID-19 because its adverse effect on solid SMEs). When we look at the jobs protected in strong SMEs (that is those SMEs that would have been expected to generate positive EBITDA otherwise), the percentage goes up to 23% of the jobs. Besides, the scheme extends the residual life of weak SMEs from 119 to 191/194 days (that is by 2 months and a half), while in the case of strong SMEs, the scheme extends life from 155 to 188/193 days (that is by 1 month). Nevertheless, the average expected residual life for those SMEs burning cash does not tell the entire story since the distribution is highly skewed. As emerge by looking at Fig. 2A, B, and C, COVID-19 has a dramatic impact on the number of SMEs that can run out of cash in 30 and 60 days.

In the ordinary scenario, we estimate that 1498 of the SMEs that are burning cash have only up to 30 days of cash to cover running costs, while additional 541 SMEs have up to 60 days. COVID-19 increases threefold the number of SMEs with less than 30 days of cash (4701) as well as those with up to 60 days (additional 1518). Besides, out of 518 SMEs with between 30 and 60 days of cash under the ordinary scenario, 510 (98%) end up with less than 30 days in the case of COVID-19. This evidence suggests that weak SMEs are hugely adversely affected by both a more negative EBITDA and reduced cash holdings because of an increase in the delinquency levels. All in all, 42% of the SMEs that have less than 30 days of cash under COVID-19 scenario are in fact SMEs that were already very weak (firms with less than 60 days of residual life in the case of ordinary scenario).

The government intervention mitigates partially the negative impact: SMEs with up to 30 days of cash decrease from 4701 to 2140 by considering CJRS only and to 1550 by considering CJRS and BBLS at 50,000 GBP. Similar reduction is obtained for SMEs with up to 60 days of cash. Thus, mitigation policies can cut up to 60% the number of SMEs that would have been expected to have less than 60 days of cash to cover their negative EBITDA in line with findings in other contexts (Block et al., 2020). However, the huge increase in the number of SMEs that move from “up to 30” days to “up to 60” days of residual life is largely ascribed to already weak SMEs.

There is no doubt about (1) the importance to help firms that are facing an unprecedented crisis and (2) the need to identify and use innovative government supporting tools (Didier et al., 2021). However, it is also very important to set up tools that can properly support firms. Our results raise doubts on the effectiveness of both UK schemes because of their non-discriminatory nature (lack of proper upfront selection): they do not discriminate according to firms’ quality, industry, or area even if research suggests that COVID-19 has different impact on different types of firm (Fairlie & Fossen, 2021; Graeber et al., 2021) and also because of its effect on consumer habits (Keane & Neal, 2021; Mehta et al., 2021; Wisniewski et al., 2021). Besides, our evidence suggests that those firms that benefit at most are, in fact, those that were already weak before COVID-19 even if this is not the strategy pursued by the UK government: sticking to the various government declaration, both schemes are not aimed at supporting weak firms, but they are supposed to protect the economic actors from an external shock. Our evidence supports the point that these schemes, by extending the life of weak firms, create more zombie firms that will not be able to trade properly when the schemes will close down (Thomas, 2021), so that the result is just postponing their bankruptcies (Schivardi et al., 2020). In other words, our results suggest that very general schemes like these ones can end up throwing good money after bad.

The doubts about the effectiveness of the scheme are further reinforced by our analysis at industry and regional level: those industries and regions that are more economically adversely affected by the COVID-19 are not those that enjoy the greatest benefit linked to the schemes, while those regions that appear to face a smaller economic negative impact related to COVID-19 are those that benefit the most by the intervention.

An effective supporting scheme should also put SMEs in a position to be able to jumpstart when the economy opens again. Currently, both CJRS and BBLS (irrespective the fact that it is called bouncing back loan scheme) do not address this aspect since the support provided targets the survival but does not provide the resources to re-start the business (e.g. build up the stock again, re-establish the link with customers). Thus, weak small and medium SMEs can even survive the period with no/or with much reduced sales just to incur the risk to fail resuming their activities because of lack of the extra resources when needed (Wiggins & Hancock, 2020). In addition, there are doubts about small firms capability to repay loans that have been largely given without any proper creditworthiness check (D. Thomas et al. 2020) raising moral hazard issues (Craig et al., 2007; Stiglitz & Weiss, 1981).

## Conclusion

How long can SMEs survive during global economic crisis triggered by COVID-19? What are the effects of the UK government intervention to help SMEs to cope with the unforeseen economic shock? To shed some empirical light on these questions, moving from the shock on sales that SMEs face, we investigate how it affects the capability of the SMEs to generate positive EBITDA using data on over 42,401 UK small and medium SMEs across 28 industries from 2016 to 2018. We also perform a robustness check by using 18,626 observation for the years 2017–2019.

We estimate earnings and then the “residual life” (days of cash available to cover the negative earnings) of the SMEs under three different scenarios: (1) ordinary scenario, expected values based on past performance and not considering any negative effect linked to COVID-19 shock; (2) scenario with the shock on sales at industry level generated by COVID-19; and (3) scenario with the implementation of the UK government policies to support small SMEs (namely, CJRS and BBLS).

We find that COVID-19 has a huge impact on small and medium firms reducing the percentage of those with positive EBITDA (that we label solid ones) from 75 to 41%. Besides, for those that are defined as weak because they are expected to generate a negative EBITDA, the days of cash available to cover the negative EBITDA goes down from 164 to 139 suggesting that COVID-19 not only increases the number of SMEs in weak position but also intensifies the speed at which cash is burned. At first sight, the government mitigation policies seem to be quite successful in supporting small- and medium-sized firms: on the one hand, the number of SMEs with positive EBITDA increases above 50%; on the other, the residual life for SMEs that burn cash increases to 183 days in the scenario of CJRS, or between 189 and 194 days in the scenario of the joint effect of CJRS and BBLS. All in all, the policies have two main effects: (1) they reduce the number of SMEs with negative EBITDA by 10%, and (2) they extend the life of those SMEs that are burning cash by 50 to 60 days. Moreover, the effect of the government policies on jobs is quite relevant: according to our estimates, the number of jobs at risk, that is jobs in SMEs with negative EBITDA, increases by around 14,000 in our sample. The implementation of CJRS allows some SMEs to turnaround and has a positive EBITDA so that the number of jobs at risk decreases by 17%. However, our analysis on the subsamples of SMEs that generate negative EBITDA irrespective of COVID-19 and those that turn to have a negative EBITDA only because of COVID-19 suggests that the SMEs that benefit the most in terms of extended days of life are those that are weak irrespective of COVID-19. This can have a very relevant negative impact later on, when firms that move from a very weak position may not be able to repay their loans and the government might be asked to step in to cover the losses of default that banks will face (Craig et al., 2007; D. Thomas et al., 2020). On the positive side, the greater number of jobs that turn to be protected are linked to those SMEs that used to be strong that is those that are expected to be in a better position to jumpstart after COVID-19. When we focus on the regional impact, we find that the biggest beneficiary of government intervention is the London area, while the hardest hit from the economic point of view are the SMEs in Scotland (lowest EBITDA and survival rate) and SMEs in Northern Ireland (with least cash days available). In terms of industry impact, we find that the biggest hit industries are agriculture and construction, while SMEs in service industry seem not to be impacted as much but they are benefiting more from mitigation policies. Again, the evidence suggests that a plain-vanilla scheme struggles to provide support to the areas that need that support the most.

All in all, our evidence suggests mixed results in terms of benefit generated by the schemes: some protection in terms of job losses but overprotection of weak firms and un-match between industries/areas that are worst hit and those that benefit at most. We ascribe the mixed results to the characteristics of the schemes that in order to be simple to use and to facilitate firms access to them in a situation of extreme emergency, are not able to effectively discriminate between solid and less solid SMEs, so that the scheme may just play the role of postponing the bankruptcy of weak firms. The lack of discrimination and the potential relatively large number of defaults on the BBLS loans from weak firms can generate a large cash outflow the UK taxpayer when they will be asked to cover the losses the banks will incur. Interestingly enough, new rounds/extension of the schemes did not introduce major changes, for instance, in terms of selection of firms that are allowed to access the schemes. In fact, this evidence raises a question that opens to further research: does the lack of proper upfront selection, by extending the life of weak economic actors and precluding the “natural selection” of firms, destroy national wealth?

Our work suffers from at least two limitations. First, we use a sample (not the entire population of UK small SMEs), and we rely on estimates in terms of small SMEs’ performance that is based on the 2016–2018 data. Thus, even if we use a relatively larger dataset, we cannot rule out that results can be different if the entire population is used. Besides, even if we perform additional test with more recent data that suggest no qualitatively different results, nevertheless, we cannot rule out that the outcomes can be different when data from the entire population including data for 2019 financial performance is used. Finally, our entire analysis is based on estimation of the current situation faced by SMEs since there are no available data. These weaknesses suggest areas for further research, namely, reproduce the analysis with larger and more updated dataset when they are available. In addition, it can be interesting to cross-check the results we obtained when historical data will be available. Second, our work is limited in terms of area covered: we explore the role of policies in the UK only, but COVID-19 is a world problem. It can be very interesting to explore and compare policies implemented by other countries. For instance, it can be interesting to compare CJRS that has been set up explicitly for the COVID-19 with similar job protection schemes available in Italy or Germany for many years and that have been marginally adjusted to accommodate the dramatic impact of COVID-19 situation. Similarly, it can be interesting to compare the situation for SMEs in the UK that implemented a moderate to rigid lockdown with the one of Italy or Spain that implemented a very strict and rigid lockdown, or with the one of Sweden that decided for a completely different strategy (no formal lockdown with strong recommendation for social distancing and reduce social activity).

Notwithstanding the limitation, our work provides a first clear evidence on the impact of the COVID-19 on the UK economy and the effect of the mitigation policies implemented by the UK government.

## Footnotes

1 More information about the scheme can be found under guidance issued by GOV.UK, entitled *Check if you can claim for your employees’ wages through the Coronavirus Job Retention Scheme*. Available at https://www.gov.uk/guidance/claim-for-wage-costs-through-the-coronavirus-job-retention-scheme

2 More information about the scheme can be found under guidance issued by GOV.UK, entitled *Apply for a coronavirus Bounce Back Loan*. Available at https://www.gov.uk/guidance/apply-for-a-coronavirus-bounce-back-loan

3 We also estimate firms with expected positive ordinary EBITDA that turns to negative. These results are available upon the requests from the authors.

## References

[CR1] Albonico, M., Mladenov, Z., & Sharma, R. (2020). *How the COVID-19 crisis is affecting UK small and medium-size enterprises.* https://www.mckinsey.com/~/media/McKinsey/Industries/Public%20and%20Social%20Sector/Our%20Insights/How%20the%20COVID%2019%20c

[CR2] Aliber, R., & Kindleberger, C. P. (2015). *Manias, panics, and crashes: A history of financial crises* (7th ed.). Palgrave Macmillan. 10.1007/978-1-137-52574-1

[CR3] Almeida, H., Campello, M., & Weisbach, M. S. (2011). Corporate financial and investment policies when future financing is not frictionless. *Journal Corporate Finance,**17*, 675–693. 10.1016/j.jcorpfin.2009.04.001

[CR4] Altman, E. (1968). Financial ratio discriminant analysis and the prediction of corporate bankruptcy. *Journal of Finance,**23*(4), 589–609. 10.2307/2978933

[CR5] Altman, E., Esentato, M., & Sabato, G. (2020). Assessing the credit worthiness of Italian SMEs and mini-bond issuers. *Global Finance Journal,**43*, 100450. 10.1016/j.gfj.2018.09.003

[CR6] Altman, E., & Sabato, G. (2007). Modelling credit risk for SMEs: Evidence from the U.S. market. *Abacus,**43*(3), 332–357. 10.1111/j.1467-6281.2007.00234.x

[CR7] Altman, E., Sabato, G., & Wilson, N. (2012). The value of non-financial information in small and medium-sized enterprise risk management. *Journal of Credit Risk,**6*(2), 95–127. 10.21314/JCR.2010.110

[CR8] Ang, J. B. (1991). Small busines uniquess and the theory of financial manegement. *Journal of Small Business Finance*, *1*, 1–13. digitalcommons.pepperdine.edu/jef/vol1/iss1/2

[CR9] Ang, J. B. (1992). On the theory of finance for privately held firms. *Journal of Small Business Finance*, *1*(3), 185–203. digitalcommons.pepperdine.edu/jef/vol1/iss3/1

[CR10] Ang, J. B., Wuh Lin, J., & Tyler, F. (1995). Evidence of the lack of separation between business and personal risk among small business. *Journal of Small Business Finance*, *4*(2/3), 197–210. digitalcommons.pepperdine.edu/jef/vol4/iss2/7

[CR11] Baker, S. R., Bloom, N., Davis, S. J., & Terry, S. J. (2020). *Covid-induced economic uncertainty.* http://www.nber.org/papers/w26983

[CR12] Bank of England. (2020). *The impact of Covid-19 on businesses’ expectations: Evidence from the decision maker panel.* (No. 2020 Q3). www.bankofengland.co.uk/quarterly-bulletin/2020/2020-q3/the-impact-of-covid-19-on-businesses-expectations-evidence-from-the-decision-maker-panel

[CR13] Bartik, A. W., Bertrand, M., Cullen, Z. B., Glaeser, E. L., Luca, M., & Stanton, C. T. (2020). *How are small businesses adjusting to COVID-19? Early evidence from a survey. National Bureau of Economic Research* (No. Working Paper 26989). http://www.nber.org/papers/w26989

[CR14] Belghitar, Y., & Khan, J. (2013). Governance mechanisms, investment opportunity set and SMEs cash holdings. *Small Business Economics,**40*(1), 49–72. 10.1007/s11187-011-9366-z

[CR15] Beltratti, A., & Stulz, R. M. (2012). The credit crisis around the globe: Why did some banks perform better? *Journal of Financial Economics,**105*(1), 1–17. 10.1016/j.jfineco.2011.12.005

[CR16] Bigelli, M., & Sánchez-Vidal, J. (2012). Cash holdings in private firms. *Journal of Banking and Finance,**36*(1), 26–35. 10.1016/j.jbankfin.2011.06.004

[CR17] Block, J. H., Fisch, C., & Hirschmann, M. (2021). The determinants of bootstrap financing in crises: Evidence from entrepreneurial ventures in the COVID-19 pandemic. *Small Business Economics.*, *forthcomin*. 10.1007/s11187-020-00445-6 PMC7829328 38624547

[CR18] Block, J. H., Kritikos, A. S., Priem, M., & Stiel, C. (2020). *Emergency aid for self-employed in the COVID-19 pandemic: A flash in the pan?,"* (No. Discussion Papers 1924). *Discussion Papers of DIW Berlin*. Berlin. 10.1016/j.joep.2022.102567 PMC9547119 36245552

[CR19] Brown, R., Rocha, A., & Cowling, M. (2020). Financing entrepreneurship in times of crisis: Exploring the impact of COVID-19 on the market for entrepreneurial finance in the United Kingdom. *International Small Business Journal,**38*(5), 380–390. 10.1177/0266242620937464 38602995 10.1177/0266242620937464 PMC7342936

[CR20] Carbó-Valverde, S., Rodriguez Fernandez, F., & Udell, G. F. (2016). Trade credit, the financial crisis, and SME access to finance. *Journal of Money, Credit and Banking,**48*(1), 113–143. 10.1111/jmcb.12292

[CR21] Casey, E., & O’Toole, C. M. (2014). Bank lending constraints, trade credit and alternative financing during the financial crisis: Evidence from European SMEs. *Journal of Corporate Finance,**27*, 173–193. 10.1016/j.jcorpfin.2014.05.001

[CR22] Cassar, G., & Holmes, S. (2003). Capital Structure and financing of SMEs: Australian evidence. *Accounting and Finance,**43*(2), 123–147. 10.1111/1467-629X.t01-1-00085

[CR23] Craig, B. R., Jackson, W. E. I. I. I., & Thomson, J. B. (2007). Small firm finance, credit rationing, and the impact of SBA-Guaranteed lending on local economic growth. *Journal of Small Business Management,**45*(1), 116–132. 10.1111/j.1540-627X.2007.00202.x

[CR24] Cressy, R. (2006). Determinants of small firms survival and growth. In M. Casson, B. Yeung, A. Basu, & N. Wadeson (Eds.), *The Oxford Handbook of Entrepreneurship* (pp. 161–193; 7). Oxford: Oxford University Press.

[CR25] Dean, T. J., Brown, R. L., & Bamford, C. E. (1998). Differences in large and small firm responses to environmental context: Strategic implications from a comparative analysis of business formations. *Strategic Management Journall,**19*(8), 709–728. 10.1002/(SICI)1097-0266(199808)19:8%3C709::AID-SMJ966%3E3.0.CO;2-9

[CR26] Didier, T., Huneeus, F., Larrain, M., & Schmukler, S. L. (2021). Financing firms in hibernation during the COVID-19 pandemic. *Journal of Financial Stability*, *53*, 100837. https://www.sciencedirect.com/science/article/pii/S1572308920301406 10.1016/j.jfs.2020.100837 PMC9761015 40478067

[CR27] Dittmar, A., & Duchin, R. (2010). *The dynamics of cash* (No. Ross School of Business Paper No. 1138). 10.2139/ssrn.1569529

[CR28] Duarte, F. D., Gama, A. P. M., & Gulamhussen, M. A. (2018). Defaults in bank loans to SMEs during the financial crisis. *Small Business Economics,**51*(3), 591–608. 10.1007/s11187-017-9944-9

[CR29] European Commission. (2003). Recommendation of 6 May 2003 concerning the definition of micro, small and medium-sized enterprises. (2003/361/EC & E. Commission, Eds.). Brussels: European Union. http://data.europa.eu/eli/reco/2003/361/oj

[CR30] Fairlie, R., & Fossen, F. M. (2021). The early impacts of the COVID-19 pandemic on business sales. *Small Business Economics*. 10.1007/s11187-021-00479-4 10.1007/s11187-021-00479-4 PMC8009687 38624577

[CR31] Fitoussi, J. P., & Saraceno, F. (2010). Europe: How deep is a crisis? Policy responses and structural factors behind diverging performances. *Journal of Globalization and Development,**1*(1), 1–19. 10.2202/1948-1837.1053

[CR32] FT Reporters. (2020, March 16). Most airlines face bankruptcy by end of May, industry body warns. *Financial Times*, p. online edition. online edition. Available at https://www.ft.com/content/30a3a26e-674f-11ea-800d-da70cff6e4d3

[CR33] Ghosal, V., & Ye, Y. (2015). Uncertainty and the employment dynamics of small and large businesses. *Small Business Economics,**44*, 529–558. 10.1007/s11187-014-9614-0

[CR34] Graeber, D., Kritikos, A. S., & Seebauer, J. (2021). *COVID-19: a crisis of the female self-employed,* (No. CEPA Discussion Papers 27). 10.25932/publishup-49810 10.1007/s00148-021-00849-y PMC8192686 34131364

[CR35] Hamilton, B. H. (2000). Does entrepreneurship pay? An empirical analysis of the returns to self-employment. *Journal of Political Economy,**108*(3), 604–631. 10.1086/262131

[CR36] Hutchinson, P. (1999). Small firms: Finance, ownership and control. *International Journal of Management Reviews,**1*(3), 343–365. 10.1111/1468-2370.00018

[CR37] Kahl, M., Liu, J., & Longstaff, F. A. (2003). Paper millionaires: How valuable is stock to a stockholder who is restricted from selling it? *Journal of Financial Economics,**67*(3), 385–410. 10.1016/S0304-405X(02)00258-1

[CR38] Kahneman, D. (2012). *Thinking fast and slow*. Penguin Books. 10.1037/h0099210

[CR39] Kahneman, D., Slovic, P., & Tversky, A. (1982). *Judgment under uncertainty: Heuristics and biases*. Cambridge University Press. 10.1017/CBO9780511809477 10.1126/science.185.4157.1124 17835457

[CR40] Keane, M., & Neal, T. (2021). Consumer panic in the COVID-19 pandemic. *Pandemic Econometrics*, *220*(1), 86–105. https://www.sciencedirect.com/science/article/pii/S0304407620302840 10.1016/j.jeconom.2020.07.045 PMC7447232 32863535

[CR41] Keynes, J. M. (1936). *The General Theory of Employment, Interest, and Money*. Palgrave Macmillan.

[CR42] Kneer, J. A. C., Horen, N. Van, Saleheen, J. N., Horen Van, C., & Saleheen, J. N. (2019). *All you need is cash: Corporate cash holdings and investment after the financial crisis* (No. No. DP14199). *CEPR Discussion Paper 14199 and Bank of England SWP No. 843.* ssrn.com/abstract=3504629

[CR43] McGuinness, G., & Hogan, T. (2016). Bank credit and trade credit: Evidence from SMEs over the financial crisis. *International Small Business Journal,**34*(4), 412–445. 10.1177/0266242614558314

[CR44] McLean, D. R. (2011). Share issuance and cash savings. *Journal of Financial Economics,**99*, 693–715. 10.1016/j.jfineco.2010.10.006

[CR45] Mehta, S., Saxena, T., & Purohit, N. (2021). The new consumer behaviour paradigm amid COVID-19: Permanent or transient? *Journal of Health Management,**22*(2), 291–301. 10.1177/0972063420940834

[CR46] Moro, A., Maresch, D., & Fink, M. (2015). Reduction in information asymmetry and credit access for small and medium- sized eneterprises. *Journal of Financial Research,**38*(1), 121–143. 10.1111/jfir.12054

[CR47] Moskowitz, T. J., & Vissing-Jørgensen, A. (2002). The returns to entrepreneurial investment: A private equity premium puzzle? *The American Economic Review,**92*(4), 745–778. 10.1257/00028280260344452

[CR48] Mramor, D., & Valentincic, A. (2003). Forecasting the liquidity of very small private companies. *Journal of Business Venturing,**18*(4), 745–771. 10.1016/S0883-9026(03)00002-8

[CR49] Myers, S. C., & Majluf, N. S. (1984). Corporate finance and investment decisions when firms have information investors do not have. *Journal of Financial Economics,**13*, 187–221. 10.3386/w1396

[CR50] ONS. (2020). *Coronavirus and the impact on output in the UK economy*. https://www.ons.gov.uk/economy/grossdomesticproductgdp/articles/coronavirusandtheimpactonoutputintheukeconomy/july2020

[CR51] Opler, T., Pinkowitz, L., Stulz, R., & Williamson, R. (1999). The determinants and implications of corporate cash holdings. *Journal of Financial Economics,**52*, 3–46. 10.1016/S0304-405X(99)00003-3

[CR52] Organisation for Economic Cooperation and Development. (2020). *Coronavirus (COVID-19): SME policy responses.* http://www.oecd.org/coronavirus/policy-responses/coronavirus-covid19-sme-policy-responses-04440101/

[CR53] Philosophov, L. V., & Philosophov, V. L. (1999). Optimization of corporate capital structure: A probabilistic Bayesian approach. *International Review of Financial Analysis,**8*(3), 199–215. 10.1016/S1057-5219(99)00018-6

[CR54] Philosophov, L. V., & Philosophov, V. L. (2005). Optimization of a firm’s capital structure: A quantitative approach based on a probabilistic prognosis of risk and time of bankruptcy. *International Review of Financial Analysis,**14*(2), 191–209. 10.2139/ssrn.403780

[CR55] Pompe, P. P. M., & Bilderbeek, J. (2005). The prediction of bankruptcy of small- and medium-sized industrial firms. *Journal of Business Venturing,**20*(5), 847–868. 10.1016/j.jbusvent.2004.07.003

[CR56] Saeed, A., Belghitar, Y., & Clark, E. (2015). Political connections and leverage: Firm-level evidence from Pakistan. *Managerial and Decision Economics,**36*(6), 364–383. 10.1002/mde.2674

[CR57] Samatas, A., Makrominas, M., & Moro, A. (2019). Financial intermediation, capital composition and income stagnation: The case of Europe. *Journal of Economic Behavior and Organization,**162*, 273–289. 10.1016/j.jebo.2018.12.033

[CR58] Schivardi, F., Sette, E., & Tabellini, G. (2020). Identifying the real effects of zombie lending. *The Review of Corporate Finance Studies,**9*(3), 569–592. 10.1093/rcfs/cfaa010 10.1093/rcfs/cfaa010 PMC7454878 40504184

[CR59] Stiglitz, J., & Weiss, A. (1981). Credit rationing in markets with imperfect competition. *American Economic Review*, *71*(3), 393–410. https://www.jstor.org/stable/1802787

[CR60] Thomas, D. (2021, March 22). UK companies in financial distress rise at fastest pace in 7 years. *Financial Times*. London (UK). https://www.ft.com/content/28d50871-2858-42e7-894d-bfde0bf5c77d

[CR61] Thomas, D., Morris, S., & Parker, G. (2020, July 26). Treasury and banks in talks to tackle coming wave of bad Covid debt. *Financial Times*. https://www.ft.com/content/aa102028-710e-43e5-a199-0198f5f16ec2

[CR62] Thomas, J., & Evanson, R. (1987). An emprical investigation of association between financial ratio use and small business success. *Journal of Business Finance & Accounting,**14*(4 Winter), 555–571. 10.1111/j.1468-5957.1987.tb00112.x

[CR63] Thorgren, S., & Williams, T. A. (2020). Staying alive during an unfolding crisis: How SMEs ward off impending disaster. *Journal of Business Venturing Insights*, *14*, forthcoming. 10.1016/j.jbvi.2020.e00187

[CR64] Walker, E. W., & Petty, J. W. (1978). Financial differences between large and small firms. *Financial Management*, *7*(4), 61–68. https://www.jstor.org/stable/3665087

[CR65] Wiggins, K., & Hancock, A. (2020, July 17). Ask and Zizzi sold with further job losses for restaurant sector. *Financial Times*, p. online edition. https://www.ft.com/content/652b1146-db7a-48f9-91b7-d68289a717ca

[CR66] Wisniewski, T. P., Polasik, M., Kotkowski, R., & Moro, A. (2021). *Switching from cash to cashless payments during the COVID-19 pandemic and beyond*. 10.2139/ssrn.3794790

[CR67] Yamori, N. (2015). Japanese SMEs and the credit guarantee system after the global financial crisis. *Cogent Economics and Finance,**3*(1), 1–18. 10.1080/23322039.2014.1002600

